# Post-Operative Thoracic Epidural Analgesia and Incidence of Major Complications according to Specific Safety Standardized Documentation: A Large Retrospective Dual Center Experience

**DOI:** 10.3390/jpm13121672

**Published:** 2023-11-29

**Authors:** Despoina G. Sarridou, Sophia Anastasia Mouratoglou, Jeremy B. Mitchell, Felicia Cox, Afroditi Boutou, Maria Braoudaki, George I. Lambrou, Maria Konstantinidou, Helena Argiriadou, Christopher P. R. Walker

**Affiliations:** 1 Department of Anesthesia and Intensive Care, AHEPA University Hospital of Thessaloniki, Aristotle University of Thessaloniki, 54124 Thessaloniki, Greece; s_mouratoglou@yahoo.gr (S.A.M.); argiriadouhelena@hotmail.com (H.A.); 2 Department of Anesthesia and Intensive Care, The Royal Brompton and Harefield Hospital NHS, Middlesex, London UB9 6JH, UK; j.mitchell@rbht.nhs.uk (J.B.M.); f.cox@rbht.nhs.uk (F.C.); 3 Respiratory Medicine Department, Hippokration Hospital, 54942 Thessaloniki, Greece; afboutou@yahoo.com; 4 Department of Clinical, Pharmaceutical and Biological Science, School of Life and Medical Sciences, University of Hertfordshire, Hertfordshire AL10 9AB, UK; m.braoudaki@herts.ac.uk; 5 Choremeio Research Laboratory, First Department of Pediatrics, National and Kapodistrian University of Athens, 11527 Athens, Greece; glamprou@med.uoa.gr; 6 Department of Respiratory Medicine, G. Papanikolaou General Hospital, 57010 Thessaloniki, Greece; markonstand@gmail.com; 7 Institute of Critical Care and Anaesthesia, Cleveland Clinic, London W1B 1LU, UK

**Keywords:** epidural analgesia, thoracic surgery, thoracotomy, adverse effects complications

## Abstract

(1) Background: Thoracic epidural analgesia is considered the gold standard in post-operative pain management following thoracic surgery. This study was designed to explore the safety of thoracic epidural analgesia and to quantify the incidence of its post-operative complications and side effects in patients undergoing thoracotomy for major surgery, such as resection of lung malignancies and lung transplantation. (2) Methods: This is a retrospective, dual-center observational study including patients that underwent major thoracic surgery including lung transplantation and received concurrent placement of thoracic epidural catheters for post-operative analgesia. An electronic system of referral and documentation of complications was used, and information was retrieved from our electronic critical care charting system. (3) Results: In total, 1145 patients were included in the study. None of the patients suffered any major complication, including hematoma, abscess, or permanent nerve damage. (4) Conclusions: the present study showed that in experienced centers, post-operative epidural analgesia in patients with thoracotomy is a safe technique, manifesting minimal, none-serious complications.

## 1. Introduction

Thoracotomy is considered to be one of the most painful surgical procedures. Severe post-operative pain after thoracic surgery can be intense and increases the risk of developing early and late post-operative complications. Early adverse effects associated with poor functional analgesia include cardiorespiratory complications including atelectasis, hypoxia, and pulmonary infection [1,2,3], while poorly managed pain may precipitate the onset of neuropathic pain post thoracotomy [4,5]. Although the development of various complications after all neuraxial blocks were extensively studied during the last decades [6,7,8,9,10], the development of post thoracotomy chronic pain remains an issue resulting in patient suffering, increased health resource utilization, and persistent post-operative opioid use (PPOU) [11]. Post-thoracotomy pain syndrome (PTPS) is a well-investigated medical entity with nearly a 10% of the patients affected developing life-changing chronic pain issues [12]. Patients with PTPS also have a high burden of neuropathic symptoms [13]. 

Apart from the medical aspects, poor pain management after major lung surgery is associated with impaired recovery, prolonged hospital stay, and increased costs, something that is a major issue even in the most developed healthcare systems worldwide. Effective and functional analgesia is the corner stone in all enhanced recovery after surgery (ERAS) protocols, especially after thoracic surgery because of its strong association with significant post-operative pain.

Thoracic epidural analgesia (TEA) is frequently regarded as the gold standard analgesic technique, as it enhances post-operative recovery, rehabilitation, and decreases post-operative morbidity and mortality without respiratory insufficiency [14,15,16]. Continuous thoracic epidural block and continuous paravertebral block were established as primary analgesic approaches for pain control following thoracotomy [17], though serratus plane blocks are increasingly used for video-assisted minimally invasive approaches. Although thoracic epidural analgesia provides a sensory block that facilitates functional analgesia, the technique itself is invasive and can cause complications or side effects.

Several previous studies proposed that paravertebral blockade is as effective and has a favorable side effect profile analgesic technique. Nevertheless, a paravertebral block may also cause complications. The proximity of paravertebral space with pleural and the sympathetic chain needs to be taken into consideration when performing this block and requires experience and advanced regional anesthesia skills, such as, of course, the TEA [18]. 

Other peripheral nerve blocks were used successfully for post-operative pain management after thoracic surgery. Intercostal nerve blocks, serratus anterior plane block SAPB, and the most recently introduced erector spinae plane block (ESPB) are nowadays well-established methods commonly associated with post-operative analgesia [19]. Cryosurgical ablation of the intercostal nerves is also undergoing a resurgence, and although thoracic epidural anesthesia is considered the gold standard, cryoanalgesia of intercostal nerves was proposed and used in the past as an alternative with quite satisfactory results [20]. 

The current trend in thoracic surgery moved towards minimally invasive surgery (including robotics); however, open procedures requiring extensive thoracotomy and intervention to the thoracic cage are still quite common. Pain is not negligible even after minimal invasive surgery where peripheral nerve blocks are regularly used in contrast to thoracic epidural [21]. The development of liposomal formulations of amide local anesthetic medicines with a duration of action of 72 h is much anticipated.

Lung transplant patients are a unique group of patients due to their co-morbid conditions. A significant proportion of these patients live with chronic pain. This includes patients with cystic fibrosis (CF) who may have CF arthropathy and chronic chest wall pain from coughing and recurrent chest infections. The association between pre-existing pain and the development of persistent post-surgical pain (PPSP) is well described [13]. One other risk factor for PPSP is the preoperative use of opioids, which are commonly used to help patients management their breathlessness whilst on the waiting list [22]. The severity of pain in patients who undergo bilateral sequential single lung transplantation (BSSLTx), especially with the clamshell incision, can be a significant factor that impairs recovery, mobilization, rehabilitation, and prolongs ICU stay. Poorly managed pain impedes functional analgesia—a satisfactory cough reduces respiratory excursion and promotes lung re-expansion. The above may precipitate pulmonary complications, in addition to the psychological and emotional impact of pain per se. Increased ICU length of stay also increases the risk of delirium, which is associated with increased mortality [23].

In the current study, we aimed to record: (a) the potential adverse events and complications associated with thoracic epidural placement, performed in lateral position in the operating room or asleep in ICU in adult patients undergoing major thoracic surgery, and (b) the incidence of major post-operative side effects of epidural anesthesia in these patients, including epidural hematoma formation, bleeding, CNS or tissue infection, and transient or permanent neurological deficits. Most importantly, we aimed to highlight and present the Trust safety standards alongside with the detailed use of documentation and incidence reporting systems, such as the electronic patient records and the risk rating matrix, which is used in our Trust patients, in the systematic assessment and identification of these adverse events, for the first time in literature.

## 2. Materials and Methods

### 2.1. Patients

After gaining institutional approval we undertook a retrospective, dual-center study of adult patients (over 18 years of age) undergoing major thoracic surgery, including lung transplantation at Harefield and Royal Brompton Hospitals, a tertiary cardiothoracic institution in London, UK. The study lasted for three and a half years and collected data involving three calendar years. We looked strictly at the period starting from January 2012 to end of December 2014 due to specific limitations on data recording and also collection. The organization and process of the data lasted an extra six-month period. The study included thoracic surgery performed with an open thoracotomy and also lung transplants and required a thoracic epidural insertion. Thoracoscopic cases were not included in the study

Each patient was assigned a code and all data were analyzed anonymously. All relevant data were collected by reviewing the medical records of patients scheduled for thoracic epidural analgesia.

All procedures and measurements were conducted in compliance with the international biomedical studies stipulations, with reference to the Declaration of Helsinki of the World Medical Association.

The trial was registered in clinicaltrials., accessed on 16 June 2023 gov under #NCT05909007.

### 2.2. Procedures

#### 2.2.1. Inclusion Criteria

Patients were included using the following criteria: >18 years of age, no active bleeding or coagulation disorders, no use of systemic anticoagulation or evidence of sepsis, and provided written informed consent after a discussion and risks and benefits.

#### 2.2.2. Exclusion Criteria

##### Patient

Patients were excluded using the following criteria: <18 years of age, active bleeding, coagulation disorders, use of systemic heparin for any reason, such as for hemofiltration (CVVHDF) after acute kidney injury, renal impairment, or systemic anticoagulation for any other reason. Additionally, in order to prevent epidural-related infection, patients with systemic inflammatory response syndrome (SIRS) or raised white blood cell (WBC) count (mostly cystic fibrosis patients) were excluded. SIRS criteria, apart from elevated temperature, tachycardia, and tachypnea, included leukocytosis with WBC > 12,000 cells/mm^3^ or <4000 cells mm^3^.

#### 2.2.3. Medical Procedures

Full blood count (FBC) and clotting screening were undertaken for all patients. Thromboelastography (TEG) was performed for all patients who were systemically heparinized for cardiopulmonary bypass (CPB), received significant blood product transfusion intra- and/or post-operatively, or were complicated with active bleeding the first post-operative hours, but still fulfilled criteria for early weaning and epidural insertion. Platelet and frozen plasma transfusion were guided by national policy and the above investigations. To minimize the risk of infection, inflammatory markers such as C- reactive protein and WBC were taken under consideration before the epidural insertion.

#### 2.2.4. Operational Procedures

Intraoperative anesthetic management involved standard AAGBI monitoring and invasive blood pressure monitoring via an arterial line. Induction to anesthesia was performed in the anesthetic room using propofol, fentanyl, and rocuronium in doses calculated based on patients’ body weight. For maintenance, sevoflurane was used in the majority of cases targeting a MAC of 2 and a BIS < 50. In all cases requiring lung isolation with one-lung ventilation, left-sided double lumen tubes were used apart from left upper lobectomies and left pneumonectomies that had indication for right double lumen tubes. Single lumen tubes with bronchial blockers were used for training purposes and in case of difficult intubation where the insertion of a double lumen tube was not possible. Flexible bronchoscopy was used routinely to confirm the correct positioning.

For elective thoracic surgery, the thoracic epidural catheter was inserted in the operating theatre either by the anesthetist under ultrasound guidance or by the operating surgeon under vision for lung resection for cancer. For elective cases, the epidural catheter was inserted with the patient awake in the majority of the cases. Asleep technique was used at the end of the surgical procedure, if necessary, when there was differentiation from the initial surgical and anesthesia plan. For lung transplant recipients, the routine practice following uncomplicated lung transplant is to insert an epidural catheter 6–24 h post-operatively. This is a few hours before the patient is weaned from mechanical ventilation, in order to ensure a safe coagulation profile and to establish a sensory block and pain relief during emergence, and subsequently for the next days. Ultrasound was occasionally used for identification of epidural space. Patients were positioned laterally having deep sedation with propofol and morphine or remifentanil infusions (asleep, lateral position). Strict full aseptic technique included surgical scrub, sterile gloves, mask, hat, and gown, and skin preparation was with 2% chlorhexidine in alcohol 70%. The usual insertion level varies between T4 and T6, and levobupivacaine 0.125% with fentanyl 2 mcg/mL was used as per the Trust protocol. Appropriate use of opioids focusing on functional analgesia was aimed for, and co-administration of opioids was avoided by prescribing plain local anesthetic solution with fentanyl PCA (patient-controlled analgesia) when needed. All procedures were conducted by experienced medical stuff. All patients had four-hourly assessments of static and dynamic pain intensity, sensory and motor block, level of sensory blockade to cold, sedation, pruritus, and observation of the epidural insertion site assessments recorded. Highly specialist pain management nurses reviewed each patient daily and adjusted analgesia to facilitate function. Sensory and motor block checks were continued for 48 after epidural removal and all patients were counseled and provided with a printed leaflet about potential adverse events e.g., abscess formation.

#### 2.2.5. Complications Evaluation

Potential major complications were categorized into three groups: epidural haematoma, infection, and neurological deficit (transient or permanent). Data were extracted from MEDICUS^®^ and ICIP^®^, London, UK, documentation programs in conjunction with the internal electronic incident reporting system (Datix^®^, London, UK) and the risk rating matrix with color-coded levels of increasing risk of patient harm (green, yellow, amber, and red). The risk rating matrix is calculated as: likelihood × consequence (L × C) scores after effectiveness of controls taken into account (Table 1). Data were prospectively recorded and retrospectively processed by the pain team of the Trust, which consisted of specialist pain nurses. The documentation and categorization of the complications and side effects were also performed by the same team. The nurses use this tool daily and it informs the frequency of patient observations, care setting, and the continuous monitoring requirement. For example, a patient who is at high to very high risk of opioid-induced ventilatory impairment (OIVI) will have more frequent sedation scoring and be nursed in a setting with continuous monitoring and a high nurse:patient ratio. Early recognition reduces the need for naloxone to reverse OIVI and the potential need for airway support. Τhe use of this tool may be time consuming as a daily practice and adds extra work while requiring extra training of the nursing staff. However, it is with no doubt a very helpful tool for the early recognition and categorization of complications and certainly contributes to good results with minimization of risks related to TEA. Other tools used for the follow-up of patients and the recording of complications and critical incidents included the institution epidural and analgesia prescription chart for assessing sensory block, motor block, epidural insertion site, vital signs, urine output, and neurological status including sedation score, in addition to infusion rate and pain intensity, pattern, and location.

#### 2.2.6. Standard Safety Protocol

The standard safety protocol includes investigations such as FBC, clotting, and septic screening with inflammatory markers such as WBC and CRP for all patients. In addiction, thromboelastography (TEG) was performed for patients with suspicion of coagulopathy and correction of deranged coagulation took place with the administration of platelets and fresh frozen plasma before any attempt for TEA insertion. In the ICU setting for lung transplant recipients, sedation with propofol was used in order to improve the conditions and eliminate the risk of nerve damage and dural puncture. Awake technique is preferred for all elective cases and full aseptic technique is used. Regular daily pain team rounds and 4 h documentation of sensory and motor block contribute to high safety standards, individualized care, and early recognition of side effects and complications. The robust use of recording and documentation systems such as MEDICUS^®^ and ICIP^®^ in conjunction with the internal electronic incident reporting system (Datix^®^) and the risk rating matrix are very useful tools in the whole process. The Standard Safety Protocol can be found in Appendix A.

## 3. Results

**Figure 1:**
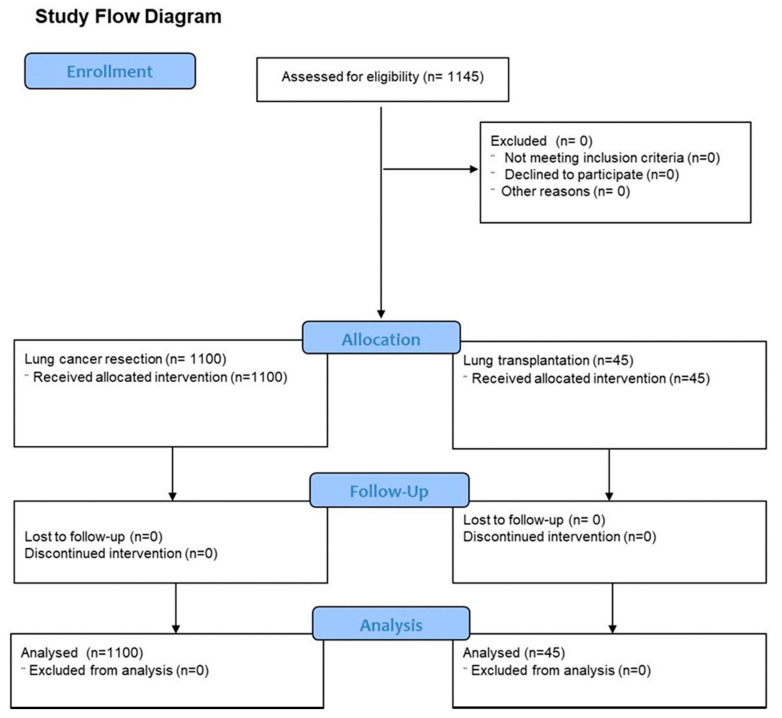
The study flow diagram for the present study.

A total of 1145 patients were included in the study, all of whom underwent major thoracic surgery and concurrent placement of thoracic epidural catheters for post-operative analgesia. In total, 1100 patients had thoracotomy, mostly for lung cancer resection, and 45 were lung transplant recipients; the majority clamshell incisions were for bilateral sequential implantation. A Consort flow diagram for the study can be seen in Figure 1.

The patient population was divided into two categories: lung cancer resection (LCR) (*n* = 1100) and lung transplantation (LT) (*n* = 45). No major complications were found. One lung transplant patient developed a subcutaneous infection at the epidural catheter site, which was treated with intravenous antibiotics. A total of 19 LCR patients had minor complications that were documented, investigated, and treated without further adverse events. These included: accidental epidural dislodgement/disconnection (*n* = 4), skin burn from hot packs (*n* = 2), skin blistering (*n* = 1) associated with the clear, occlusive adhesive dressing, inappropriate strong opioid co-administration (*n* = 6), and less significant incidences such as shoulder pain designated as other complications (*n* = 6). The observed complications are summarized in Table 2.

The distribution of patients was 96.07% for LCR and 3.93% for LT patients. A total of 95% of the patients with complications (incidence 1.75%) belonged to the LCR group of patients. Of note, LCR patients composed 1.66%, whilst LT patients formed 0.09% of the total population. In addition, accidental epidural dislodgement/disconnection was observed in the 0.35% of LCR patients, skin burn was observed in 0.17% of LCR patients, skin blistering was observed in 0.09% of LCR patients, and inappropriate strong opioid co-administration was observed in 0.52% of LCR patients. Finally, 0.09% of LT patients were observed with infections. Within the population without complications, 96.09% came from the LCR patients and 3.91% came from the LT patients. Within the total population, 94.41% from LCR patients had no complications and 3.84% from LT patients had also no complications. No other serious complications were observed. The number of complications was small relative to the large number of thoracic epidurals placed within the institution (1.75%). An established system of early recognition and reporting of adverse events contributes to the above.

## 4. Discussion

In this retrospective dual-center study, we aimed to evaluate the incidence of both major and minor complications in patients undergoing epidural anesthesia for major thoracic surgery following a strict, standardized safety protocol, and also the clinical utility of electronic recording methods and tools such as the risk rating matrix, in order to identify the incidence of these complications.

Thoracic epidural placement in the anesthetized patient can be a safe central neuraxial block technique in experienced hands when safety protocols concerning bleeding, infection, and nerve injury are followed. Thoracic epidural analgesia remains the gold standard for post-operative pain management following lung resection and lung transplantation. The risk of major complications is ever-present and is likely to be increased in patients with an exaggerated inflammatory response, SIRS, immunosuppressive regimes, or disordered coagulation. These may occur post-operatively in this patient population. Neurological damage is also a potential complication and may be transient or permanent. The establishment of increased safety standards across the hospital sites reduced the above risks. Our data of at-risk patients demonstrate the safety and efficiency of this analgesic technique. These results are in line with other studies, where no significant complications were observed with the exception of hypotension [17]. Another report demonstrated that complications such as hypotension, bradycardia, atelectasis, and need for ICU were observed but at a lower frequency in thoracic epidural analgesia compared to lumbar epidural analgesia [24]. A review of lung cancer surgical patients suggested that complications did occur following epidural analgesia, but there were no significant differences when comparing epidural with subpleural analgesia [25]. Lung atelectasis may be present following thoracotomy and may develop into pneumonia due to retention of secretions [26,27,28]. Pain prevents effective coughing, deep breathing, and a patient’s likelihood of completing physical therapies post-operatively. Poorly controlled post-operative pain can increase surgical morbidity and may also lead to chronic pain and post-thoracotomy pain syndrome [29,30,31].

A broad range of insertion techniques exists, and anesthesia physicians may site epidural catheters with the patient asleep (in a lateral position or awake (sitting) using a midline or paramedian approach). Application of robust safety standards and minimization of potential complications result in safe and good practice. The establishment of a specific pathway allows complications and critical incidents to be identified early. They can be reported and treated sooner, and this supports the process of continuously improving the quality of patient care.

Thoracic epidural infusions of local anesthetic solutions (with or without opioids) are the principle mode providing post-operative analgesia for major thoracic surgery and are considered to be a very good choice in post-operative pain management [32]. Other studies showed that this technique has several benefits, such as decreased cardiovascular pulmonary and gastrointestinal morbidity and mortality [33,34,35,36]. Some consider epidural analgesia to be invasive, labor-intensive, and expensive, yet the costs and potential risks are considered justified because of the assumed cost-to-benefit ratio. Previous studies also showed a shorter length of hospital stay after major abdominal/thoracic surgery. Thus, considering cost-effectiveness to aforementioned advantages, it can be said that this procedure is appropriate for post-operative pain management. However, several studies found that epidural analgesia may have disadvantages, suggesting less optimistic results. These may be considered when it is used in conjunction with minimally invasive surgical techniques, fast-track post-operative rehabilitation strategies, and the widespread use of prophylactic anticoagulant regimens. Other factors influencing its use include the availability of less invasive but equally effective alternative regional analgesic techniques, the difficulty of performing detailed local audits that would provide risk–benefit data, and litigation concerns.

Neurological complications, either permanent or transient, remain perhaps the most significant complications related to TEA. Paraplegia, although quite rare, is a recognized complication, especially where thoracic epidural is performed primarily under general anesthesia. Therefore, the risk of paraplegia remains present with all its catastrophic consequences. Moreover, in the thoracic level, the insertion line is in close proximity to the costovertebral angle, which increases the risk of nerve damage [4]. In addition, puncture of epidural vessels during catheter insertion occurs during 3–12% of attempts. However, the subsequent formation of a haematoma causing neurological injury is a rare complication [37].

According to some studies, the incidence of chronic post-thoracotomy pain could rise up to 50% and can radically evolute in terms of severity and disability to 5% of overall cases. The surgical technique is a significant factor affecting the development of post-thoracotomy pain. Several studies in the past attempted to associate the appearance of post-thoracotomy pain with different surgical techniques and incisions and also compare them. Even though video-assisted thoracotomy surgery (VATS) is generally considered less invasive than open techniques and reduces the levels of immediate post-operative pain, the benefits of it in terms of chronic pain are questionable from several studies, as on the long term it looks as if the development of chronic pain is not unavoidable and follows similar rates to the ones after open thoracotomy. Moreover, the relatively bigger sizes of trocars used for VATS procedures are sinister for chronic pain development. In addition, intercostal nerve damage is strongly associated with neuropathic pain and is unavoidable both on rib spreading and closure as well. Therefore, preservation of intercostal nerves is suggested. At the same time, muscle sparing procedures appear to offer a benefit over muscle cutting approaches, although the comparison between rib resection versus no rib resection remains quite controversial [5].

Complications related to thoracic epidural when occurring can be catastrophic and life threatening, and may result to longer hospital stay, whilst increasing morbidity and even mortality. Permanent nerve damage, which can take place in 1:10,000 patients, should always be considered and mentioned during the anesthetic pre-assessment phase and also documented on the consent form. Other major complications include subarachnoid block, cardiorespiratory depression, and local anesthetic toxicity. Published literature also identified several minor complications (side effects) of thoracic epidural anesthesia including hypotension, pruritus, post-operative nausea and vomiting, accidental removal of the catheter, local inflammation of the insertion point, and accidental disconnection of the catheter [6].

Some other studies suggest that the role of regional techniques becomes questionable. A study on paediatric population revealed no superiority of locoregional techniques compared to solely general anesthesia and standard pain relief on the development of chronic post-thoracotomy pain and that its incidence in general is relatively low [38]. In a different context, another study that investigated the prevalence of chronic post-thoracotomy pain in adults who underwent thoracic surgery in childhood or youth showed that the risk is lower if the surgery is performed in young age and the pain is of neuropathic etiology more than anything else [39].

Symptoms related to the formation of epidural abscess can manifest as localized tenderness, back pain, and in more advanced stages with systematic infection and sepsis. Alongside the aforementioned conditions, neurological symptomatology can also be found when compression of nerves occurs. Conservative treatment can be an option, but sometimes decompression and care in a specialized neurosurgical unit are required. On a similar but different note, active bleeding coagulopathy and cardiopulmonary bypass precipitate for increased risk of epidural hematoma. However, most cases in the literature were linked with the catheter removal. Most studies suggest the implementation of clear protocols with regular neurological examination and regular acute pain team rounds in order to detect neurological deficits in the early stage [7].

In a similar context, epidural hematomas are quite rare, but may lead to disastrous outcomes. Platelet count and clotting times are necessary investigations and also thromboelestography or thromboelastometry could be useful tools. The correction of coagulation disorders is without any doubt necessary, especially for the cases of post-operative insertion of TEA in ICU. A platelet count of more than 80.000 and clotting times within range are recognized as basic safety standards before any attempt for an epidural catheter insertion [6].

A modern regional anesthesia technique with promising results is the bilateral continuous serratus anterior blockade, which can be used for post-operative analgesia after bilateral sequential lung transplantation [40]. In another study, rhomboid intercostal and subserratus (RISS) plane block were used for analgesia also for lung transplantation [41]. In a study by Isaza et al., intercostal nerve cryoanalgesia was compared with thoracic epidural analgesia in patients undergoing lung transplantation. This study demonstrated that opioid consumption was similar to both groups as well as the pain intensity scores (but limited by no long-term follow-up). Interestingly, the requirements for rescue analgesia (lidocaine transdermal plaster as per their protocol) were lower in the cryoanalgesia group. Additionally, even though the cryoanalgesia cohort was free from complications, four patients from this group subsequently needed thoracic epidural for pain relief. The need for an invasive neuraxial intervention for rescue analgesia is indeed a significant adverse event. Lastly, 2 patients from the 43 included in the thoracic epidural cohort developed significant haemodynamic instability after the first test dose, which was treated successfully [42].

Another retrospective STROBE-compliant cohort study investigated the impact of surgical technique and the analgesia outcomes again on lung transplant patients. Thoracotomy was associated with higher pain intensity scores than sternotomy and clamshell incision. Epidural analgesia was given in total to 168 patients in ICU post-operatively when systemic opioids were not enough to achieve adequate pain cover. No epidural haematoma or abscesses were diagnosed, and two complications involved dural tap with mild headache and suboptimal sensory block due to low insertion level, requiring repositioning of the catheter. So, in terms of safety, TE was an efficient uncomplicated method of analgesia on the above cohort [43].

Comparative studies between thoracic epidural analgesia and erector spinae block for thoracic surgery are limited in the literature. However, there are a few recent studies involving patients undergoing liver surgery. The place of TEA in ERAS is, however, questionable from many studies that promote the use of other less invasive regional anesthesia techniques. A recent study included 50 patients for liver resection who received TEA or ESPB for liver resection. The analgesic effect appeared superior to the TEA cohort [44]. On the opposite, another study on adult donors for hepatectomy compared ESPB and thoracic epidural for post-operative analgesia and revealed superiority of ESPB in terms of opioid consumption, pain scores, and mean lung volume (MLV). In addition, ESPB had an enhanced safety profile [45].

A related meta-analysis, which attempted to compare different analgesia methods, for thoracoscopic surgery though, was also quite interesting. A total of 35 trials were included, and comparison between thoracic epidural, paravertebral block, deep and superficial serratus anterior plane blocks, erector spinae plane block, and intercostal block took place. Superiority of TEA and PVB was noted in terms of analgesic effect; however, the TEA showed higher incidence of pruritus, while PVB had lower rates for coagulation and puncture complications [46].

There is definitely a lack of comparative randomized double-blind studies between TEA and more modern regional anesthesia techniques such as erector spinae block and serratus plane block, which perhaps are the ones that are more popular. Additionally, in most of the aforementioned studies, no clear mention was noted on safety standards, the actual TEA insertion technique per se, and the use of systems for data recording as well. From this point of view, further research is required in this field, focusing especially on the complications, adverse effect rates, and also the times for anesthesia preparations. Another limitation is that our study is a retrospective one, and the lung transplant patients’ cohort was limited in numbers; therefore, comparison between the two groups was not possible. Additionally, the relatively small sample size of 1100 cases in conjunction with the very low risk of < 0.01 related to TEA creates another limiting factor to the study. However, the strengths of the study included the use of electronic records and systems in order to reduce the possibility of missing values and also the same protocol of the study used crossed the site permissive of a large number of patients enrolled. An additional plus was the fact that asleep epidural technique was used in the lung transplant recipient population, for which relevant data are lacking from the literature.

The efficacy of thoracic epidural as an anesthetic technique is accepted and established, but how can we really safeguard uncomplicated procedures and good practice for epidural catheter placement in anesthetized patients including the lateral position on an intensive care unit (ICU) bed?

## 5. Conclusions

The present study demonstrated that post-operative epidural analgesia in a large cohort of thoracotomy patients was associated with few complications, none of which are serious. The inpatient pain clinical nurse specialists are very proactive and surveil patients receiving thoracic epidural analgesia, with nurses in all clinical areas required to have an education and to complete competency-based assessments for this analgesic technique. Moreover, detailed electronic recording and utilization of tools, such as the risk rating matrix, enhance close monitoring of potential complications. Education and training of ward nursing staff and the presence of clinical nurse specialists with expertise in the assessment and management of neuraxial analgesia contributed significantly to the safety and efficacy of perioperative analgesia and enhanced patient safety by regular surveillance. Apart from the lung cancer resection cohort, which showed good results, the lung transplant recipients treated with the local protocol as described above, had uncomplicated post-operative analgesia with thoracic epidural. TEA may promote ERAS strategies after major thoracic surgery when safety standards and minimization of adverse effect rates and major complication take place.

## Figures and Tables

**Table 1:** The risk rating matrix used to identify the likelihood of complications.

		Consequence				
		Insignificant (1)	Minor (2)	Moderate (3)	Major (4)	Catastrophic (5)
**Likelihood**	Almost certain (5)	Medium (5)	High (10)	High (15)	Very high (20)	Very high (25)
	Likely (4)	Low (4)	Medium (8)	High (12)	Very high (16)	Very high (20)
	Possible (3)	Low (3)	Medium (6)	Medium (9)	High (12)	High (15)
	Unlikely (2)	Low (2)	Low (4)	Medium (6)	Medium (8)	High (10)
	Rare (1)	Low (1)	Low (2)	Low (3)	Medium (4)	Medium (5)
Consequence Descriptors						
5	Catastrophic	One or more fatalities or severe irreversible disability to one or more people				
4	Major	injury or impairment to one or more persons; major injury leading to long-term incapacity/ disability; lost injury time >14 days				
3	Moderate	Short term disability to one or more persons; lost injury time 4–14 days				
2	Minor	Minor injury or illness with minor medical treatment; lost injury time 1–3 days				
1	Insignificant	First aid or minor medical treatment				

**Table 2:** Summary of observed complications in LCR and LT patients after epidural post-operative analgesia.

Administration	n
Epidural dislodged/disconnected accidentally (includes 1 × confused patient pulled hers out—yellow)	4
Skin blistering under insertion site dressing (amber)	1
Skin burn from hot pack use on shoulder (yellow)	2
Paravertebral catheter mislabelled as epidural	1
Transfer of patient from level 1 to level 2 care facility because of oliguria requiring	1
**Inappropriate Strong Opioid Co-Administration**	**n**
Strong oral or intravenous opioids administered concurrently with opioid containing TEA ^1^	5
Plain local anesthetic infusion prescribed but opioid containing TEA infusion used	1
**Medicines Management/Communication/Patient Care/Other**	**n**
Failure to discard opioid containing TEA infusion once disconnected from patient	1
Lack of patient observations after premature cessation of TEA infusion as reporting dysesthesia	1
Failure by anesthetists to hand over presence of TEA catheter to PACU staff	1
No TEA infusion device available so PCA pump used	1
Complications to total number of operations ratio:	0.017

^1^ TEA: thoracic epidural analgesia.

## Data Availability

Data are contained within the article.

## Appendix A. Standard Safety Protocol

**Table:**

Investigations	Basic Points	Suggested Management
FBC	WBC, Platelet count	PLT transfusion
Clotting screening	INR, aPTT, PT	Correction of coagulopathy
Septic profile	WBC, CRP	If raised avoid TEA
TEG	Deranged parameters	Correction of coagulopathy. Consider FFP, cryo, etc.
**Theatre setting**	Elective cases for lung cancer resection Conversion to open thoracotomy insertion at the end of the procedure before awakening	Awake full aseptic technique for all elective cases Asleep full aseptic technique–sedation with propofol
**ICU setting**	Asleep technique before weaning Lung transplant patients	Asleep full aseptic technique–sedation with propofol
**Nursing care**	Specialist nurses Pain team Pain consultant	Education and training Clinical examination Documentation Recording Reporting
Daily ward rounds	High vigilance for any related issues	Adjustment of analgesia 48 h observations after TEA
Neurological assessment	Signs of new neurological deficit	4-hourly assessment of sensory and motor block
Individualized care	Early recognition of side effects and complications	Treatment as per the trust protocols
**Reporting and documentation systems** **risk rating matrix**	Documentation and reporting of side effects and complications Color coded system of risk categorization	Documentation systems: MEDICUS^®^ ICIP^®^ Internal electronic incident reporting system: Datix^®^ Close observation on patients with high risk for complications
